# Nanoparticle-based immunotherapy of pancreatic cancer

**DOI:** 10.3389/fmolb.2022.948898

**Published:** 2022-08-29

**Authors:** Gaetan Aime Noubissi Nzeteu, Bernhard F. Gibbs, Nika Kotnik, Achim Troja, Maximilian Bockhorn, N. Helge Meyer

**Affiliations:** ^1^ University Hospital of General and Visceral Surgery, Department of Human Medicine, University of Oldenburg and Klinikum Oldenburg, Oldenburg, Germany; ^2^ Department of Human Medicine, University of Oldenburg, Oldenburg, Germany

**Keywords:** pancreatic cancer, tumor microenvironment, nanoparticles, immune checkpoint inhibitors, immunotherapy

## Abstract

Pancreatic cancer (PC) has a complex and unique tumor microenvironment (TME). Due to the physical barrier formed by the desmoplastic stroma, the delivery of drugs to the tumor tissue is limited. The TME also contributes to resistance to various immunotherapies such as cancer vaccines, chimeric antigen receptor T cell therapy and immune checkpoint inhibitors. Overcoming and/or modulating the TME is therefore one of the greatest challenges in developing new therapeutic strategies for PC. Nanoparticles have been successfully used as drug carriers and delivery systems in cancer therapy. Recent experimental and engineering developments in nanotechnology have resulted in increased drug delivery and improved immunotherapy for PC. In this review we discuss and analyze the current nanoparticle-based immunotherapy approaches that are at the verge of clinical application. Particularly, we focus on nanoparticle-based delivery systems that improve the effectiveness of PC immunotherapy. We also highlight current clinical research that will help to develop new therapeutic strategies for PC and especially targeted immunotherapies based on immune checkpoint inhibitors.

## Introduction

Pancreatic cancer (PC) is a highly aggressive cancer, with an average survival of about only 15 months and only 6%–8% of patients diagnosed with PC are still alive after 5 years. This is mainly because PC is highly invasive in nature and the diagnosis is often made at a very advanced stage of the disease (Cai et al., 2021; Wu and Cai, 2021). In addition—despite decades of extensive (clinical) research—the key biological aspects of rapid PC progression, metastasis and therapy resistance remain obscure (Effenberger et al., 2012; Wang et al., 2021). Furthermore, the rare and complex tumor microenvironment (TME) of PC is typically characterized by a desmoplastic stroma that accounts for more than 70% of the tumor volume and is predominantly composed of stromal cells and extracellular matrix (ECM). Stromal cells in PC mainly comprise endothelial cells, cancer-associated fibroblasts (CAF) and immune cells. The ECM includes laminin, glycosaminoglycan, integrin, matrix metalloproteinase (MMP) and collagen (Murakami et al., 2019). Due to its dense nature the stroma modulates numerous features of the TME such as angiogenesis, ECM deposition, and inflammatory responses which consequently contribute to poor treatment outcomes and patient survival (Chan-Seng-Yue et al., 2020).

Poor efficiency of chemotherapy in the treatment of PC is mainly attributed to the density of the TME, which does not allow drugs to sufficiently penetrate into the tumor tissue (Schizas et al., 2020). In this regard, therapy resistance is primarily a result of the pressure generated by the desmoplastic stroma on the blood and lymph vessels surrounding PC, resulting in low blood flow, hypoxia, and limited exchange of molecules. In addition, pancreatic stellate cells (PsC), hyaluronan, osteonectin and other factors such as angiostatin and endostatin present in the stroma slow down drug delivery. Soluble components of the TME, including various cytokines and growth factors, further enhance the crosstalk between the dense stroma and tumor cells. This eventually leads to atypical and widespread fibrosis and accumulation of ECM proteins associated with tumor growth and density development of the PC microenvironment (Bazzichetto et al., 2020; Moon et al., 2020). These include insulin-like growth factor, transforming growth factor *β* (TGF-β) and tumor necrosis factor *α* (TNF-α) which contribute to low drug delivery by promoting proliferation of PsC and stimulating ECM synthesis (Chan-Seng-Yue et al., 2020; Du et al., 2020).

The presence of various immune cells, both of the adaptive and innate immune system, as well as various immunoregulatory molecules which mainly exert immunosuppressive activity, further contribute to the complexity of the TME in PC. This facilitates immune evasion and thus promotes tumor progression (Ren et al., 2018; Wu and Cai, 2021). The immunosuppressive cells in the TME of PC are mainly tumor associated macrophages (TAMs), myeloid-derived suppressor cells (MDSCs), and tumor-infiltrating lymphocytes (TILs), which are composed of CD4^+^ T cells, CD8^+^ T cells, and FOXP3^+^ regulatory T cells (Tregs). TILs play an important role in the response to treatment, in which the presence of CD4^+^ and CD8^+^ T cells predicts increased survival whereas the elevated presence of FOXP3^+^ Tregs predict poorer outcomes (Ino et al., 2019; Murakami et al., 2019). Furthermore, other immune cells present in the PC microenvironment, such as neutrophils and natural killer (NK) cells also potentially display pro- or anti-tumor functions, respectively, and thus may contribute to survival outcomes (Huber et al., 2020).

Overall, the peculiarities of the TME in PC result in diminished oxygenation, desmoplastic stroma and eventually the spread of an immunosuppressive cancer, which facilitates treatment resistance to immunotherapy, targeted tumor therapy and chemotherapy (Murakami et al., 2019; Karamitopoulou, 2020). Recently, the use of immune checkpoint inhibitors (ICIs) and the development of cancer vaccination to reactivate the immune system in malignant diseases has led to a revival of cancer immunotherapy and shown promising results (Schizas et al., 2020). However, attempts to develop efficient PC-specific immunotherapy have fallen short of expectations. As with chemotherapy, the TME has been identified as a major bottleneck in immunotherapy too. Therefore, it is necessary to develop immunotherapeutic strategies that are able to penetrate the PC TME (Das et al., 2019; Fan et al., 2020). With regard to targeted delivery, nanoparticles have generally shown promising results in cancer therapy. In addition, nanoparticles have a long residence time and potentially minimal side effects and toxicities (Yang et al., 2021), thus possibly avoiding (immune) therapy-resistance of PC.

Nanoparticles (NPs) are nanometer-sized materials that have characteristics similar to biomolecules and can be modified to carry molecules to different biological sites and promote their interactions (Kim et al., 2010). An inherent property of NPs is their large surface to volume ratio, allowing their surface to be covered with several thousands to millions of drug molecules (Khan et al., 2019). NPs used for drug-delivery can be divided into three main groups: lipid-based, inorganic and polymeric. Lipid-based NPs (LNPs) are widely used in biomedicine due to their simple formulation, biocompatibility and bioaccessibility. As a result, therapeutic LNPs have been frequently approved by the FDA with liposomes being the most widely used. Inorganic NPs are often metal-based and include, amongst others, gold NPs and iron oxide NPs. Inorganic NPs are characterized by variable physical, electrical, magnetic and optical properties. For example, magnetic iron oxide NPs made of maghemite (Fe_2_O_3_) or magnetite (Fe_3_O_4_) have supermagnetic characteristics and have been successfully used as contrast molecules in magnetic resonance imagining (MRI) and as drug carriers. Recently, calcium ion nanomodulators based on CaCO_3_ or (Ca_3_(PO_4_)_2_) NPs have been developed for mitochondria-targeted multimodal cancer therapy (Zheng and Ding, 2022). It was shown that the combination of the PEG-coated curcumin-loaded calcium carbonate nanomodulator, ^PEG^CaCUR, with ultrasound treatment induced robust immunogenic cell death (ICD) in a tumor-xenografted mouse model. Enhanced ICD was attributed to an increase in mitochondrial Ca^2+^ overload, along with subsequent upregulation of reactive oxygen species (Zheng et al., 2021). Polymeric NPs can be derived from natural or synthetic components, either monomers or preformed polymers. Their main characteristic is their ability to allow large formulations, that ensure adequate drug delivery. Due to their ability to deliver drugs to targeted sites at optimal doses, increased half-life in the blood, reduced toxicity and resistance to degradation by endogenous enzymes, NPs have attracted great interest in medicinal and pharmaceutical applications.

Overall, the use of NPs could address many of the weaknesses of modern anti-cancer therapy (Park et al., 2018; Khan et al., 2019; Mitchell et al., 2021). Comprehensive overviews on nanoparticle-based immunomodulatory strategies and their application in the therapy of various diseases including (metastatic) cancer, have already been provided elsewhere (Feng et al., 2019; Yu et al., 2020; Zhang et al., 2021). In the first part of the present review we summarize the challenges of immunotherapy specific to PC, briefly evaluating the shortcomings that could potentially be compensated using modern nanomedicine. In the second part we discuss clinical application of nanoparticle-mediated targeting in PC immunotherapy including an overview of current developments in NP-based drug delivery of immunomodulators and clinical trials in the field of NP-based immunotherapy.

## Article types

Narrative review.

## Challenges of pancreatic cancer immunotherapy

As described above, PC is characterized by its unique TME which is rich in stromal components also including myofibroblast, immune cells and growth factors that act as barrier and contribute to drug evasion (Feig et al., 2012). While chemotherapy has only shown moderate success and is associated with high toxicity (Marsh Rde et al., 2015; Christenson et al., 2020), many studies have been conducted on novel immunotherapeutic strategies, including ICIs, monoclonal antibody (mAb) therapy, adoptive cell transfer (ACT) including chimeric antigen receptor (CAR) T cells, vaccines, and other forms of immunotherapy. Potentially, PC immunotherapy could improve anti-tumor responses, decrease the immunosuppressive effects in the TME and, as a result, increase survival (Fan et al., 2020; Principe et al., 2021). However, despite exceptional results being observed in the immunotherapy of various other solid cancers such as melanoma, PC immunotherapy has fallen short of expectations thus far.

### Immune checkpoint inhibitors

**FIGURE 1:**
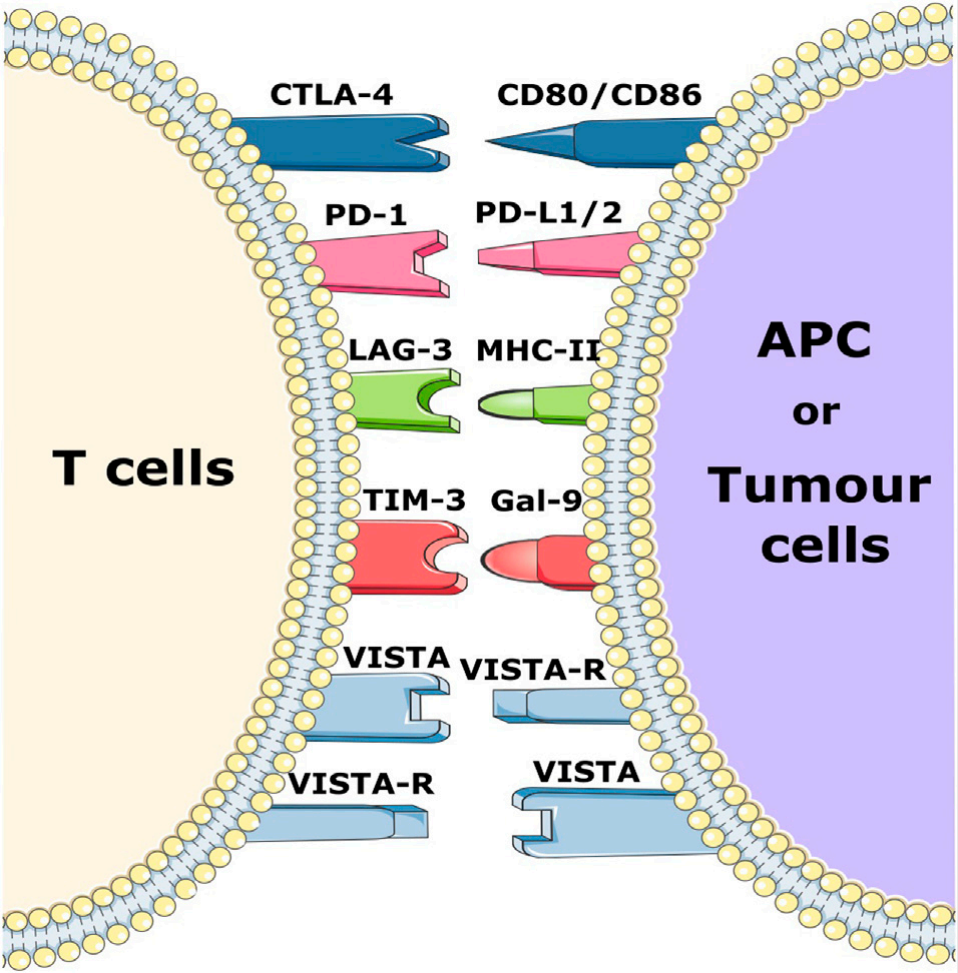
Schematic representation of various immune checkpoint receptors and their respective ligands. Immune checkpoint molecules expressed on T cells bind to their respective ligands on APCs and/or tumor cells and trigger a negative or positive signal to T cells.

Tumor cells can exploit various negative immune checkpoint regulators (NCRs) expressed on different infiltrating cells present in the pancreatic TME in order to evade the immune response (Figure 1). For example, cytotoxic T lymphocyte-associated antigen 4 (CTLA-4), an NCR which, in addition to CD8^+^ cytotoxic T cells, is also expressed on Tregs. It displays high affinity to CD80 and CD86 and widely contributes to immunosuppression. In addition, programmed cell death 1 (PD-1) and PD-ligand 1 (PD-L1) play an important role in tumor immune evasion by blocking the activation of cytotoxic T cells (Johansson et al., 2016; Fan et al., 2020).

In recent decades, therapies based on the blockade of NCRs with blocking antibodies (Ab), often referred to as checkpoint inhibition, have been used to treat solid tumors such as melanoma and lung cancer. Blockade of NCRs or their ligands reactivates immune cells and increases the anti-tumor efficacy of the immune system (Emens and Middleton, 2015; Fan et al., 2020). Since the discovery of NCRs, many of these ICIs such as ipilimumab and tremelimumab (CTLA4 inhibitors), durvalumab and atezolizumab (PD-L1 inhibitors) as well as pembrolizumab and nivolumab (PD-1 inhibitors) have been successfully applied in the treatment of solid tumors (Chi et al., 2020; Principe et al., 2021). Monotherapeutic application of these checkpoint inhibitors in PC, however, did not show any promising results in clinical studies thus far (Li et al., 2021). Other clinical trials in phase I/II with atezolizumab in combination with other drugs are currently recruiting patients (NCT03829501 and NCT04820179) (Schizas et al., 2020). Furthermore, there is increasing research on other NCR molecules including the V-domain Ig-containing suppressor of T-cell activation (VISTA), lymphocyte activation gene 3 (LAG-3) and T-cell immunoglobulin and mucin-domain containing-3 (TIM 3) (Li et al., 2021). LAG-3 is expressed primarily on NK cells, B cells, CD4^+^, and CD8^+^ T cells, where it blocks T cell receptor signaling by binding with high affinity to MHC class II molecules, thereby decreasing anti-tumor T cell activity. LAG-3 has been found to be expressed on tumor-infiltrating T cells in PC, and its expression is associated with poor disease outcomes Consequently, clinical studies are investigating the inhibitory effect of LAG-3 in solid cancers (Maruhashi et al., 2020; Li et al., 2021; Seifert et al., 2021).

In PC, TIM-3 levels are much higher than in normal tissues, and its overexpression is correlated with more rapid disease progression, highlighting its immunosuppressive role. In addition, its ligand galectin-9 is known to induce the production of human leukocyte antigen B associated transcript 3 (BAT-3), which inhibits T cell function and promotes apoptosis. A phase I-II clinical trial against advanced solid tumors with a combination of anti PD-1 and anti-TIM-3 (NCT03744468) is currently recruiting patients (Peng et al., 2017; Popp et al., 2021).

Several studies have already demonstrated the importance of VISTA in PC (Blando et al., 2019; Hou et al., 2021). As a member of the B7 family and PD-L1 homologue, VISTA is expressed in PC, endothelial cells and T cells. This expression inhibits the effector functions of T cells by preventing the release of granzyme B from cytotoxic T cells as well as inhibiting the release of IL-2 from CD4^+^ T cells (Yasinska et al., 2020; Noubissi Nzeteu et al., 2022). Macrophages also express high levels of VISTA and are associated with the PC microenvironment where they are involved in the immunosuppressive effect of the TME (Blando et al., 2019). Furthermore, a significant decrease of metastatic PC nodules was observed after inhibition of VISTA in mouse models with liver metastasis (Hou et al., 2021). VISTA blockade is currently being tested in phase I clinical trials for the treatment of advanced and metastatic tumor disease (NCT05082610 and NCT04475523). Taken together, it is clear that blocking VISTA could be a potential immunotherapeutic strategy in PC.

Although immune checkpoint inhibition beyond conventional CTLA-4 or PD-1 blockade undeniably has substantial potential in PC treatment, it will remain difficult to reverse immunosuppression based on ICI monotherapy strategies alone. This is also attributed to the immune-related adverse effects (irAEs), which significantly contribute to late systemic toxicity. Unfortunately, irAEs appear to be largely dose-independent, thus lower doses do not necessarily reduce their adverse effects (de Miguel and Calvo, 2020). Additionally, ICI therapy is implicated with hyperprogression of the disease. In order to overcome these limitations, targeted strategies need to be developed. Here, targeted NP-delivery of ICIs has great potential at reducing irAEs (Li et al., 2021; Yang et al., 2021).

### CAR T cells

T cells are the major immune cells that display anti-tumor activity. It is therefore crucial to reactivate tumor-infiltrating T cells to facilitate clearance of tumor cells. Promising research has been conducted in recent years on adoptive cell therapies (ACTs), which include the infusion of genetically modified T cells for the treatment of solid tumors: CAR T cell or TCR-modified T cell therapy. CAR T cell therapy has successfully improved the prognosis of patients with follicular lymphoma, mantle cell lymphoma and relapsed/refractory large B-cell lymphoma (Haydu and Abramson, 2021). However, in solid cancers the low proliferation and persistence of CAR T cells due to immune checkpoints such as the PD-1/PD-L1 pathway and physical barriers present in the TME, which slow CAR T cell penetration, is detrimental for the outcome of CAR-T cell therapy (Marofi et al., 2021). Strategies have been suggested to improve the efficacy and facilitate the delivery of CAR-T cells in solid cancers, one of which is the use of stimulator of IFN genes (STING) agonists. In a mouse model of breast cancer STING agonists yielded a higher anti-tumor activity and persistence of CAR-T cells. However, tumor regression was only observed when a PD-1 inhibitor was added (Sterner and Sterner, 2021; Xu et al., 2021).

Optimization of the target antigen is critical for the success of CAR T cell therapy. Mesothelin (meso) is a glycoprotein that is highly expressed in multiple cancers, including lung and PC, and is associated with degenerative disease outcomes. It is involved in cancer invasion and progression as well as cell adhesion. Six patients were treated in a phase I trial to assess the safety and efficacy of meso-CAR T cells. Two patients showed disease stability, with a progression free survival of 3.8 and 5.4 months, three patients had a stabilized metabolic active volume (MAV), while one patient experienced a reduction of 69.2% in MAV. (Beatty et al., 2018; Ali et al., 2019). Two clinical studies targeting mesothelin with CAR T cells (NCT04809766 and NCT03638193) are currently recruiting.

Nanotechnology is already widely used to stimulate the expansion of CAR T cells both *in vivo* and *in vitro* (Wang et al., 2020a) and nanoparticle-based delivery of immunomodulators has the potential to overcome immunosuppression in the TME and increase the effectiveness of CAR T cell therapy (Balakrishnan and Sweeney, 2021). However, despite the potential shown in pre-clinical models, it is not clear whether these approaches can surmount the considerable challenges posed in PC in terms of the physical exclusion of infiltrating T cells and their deactivation by the immunosuppressive PC TME.

### Cancer vaccination

Cancer vaccines are currently being considered as a strategy to circumvent the aridity of PC treatment by promoting stimulation of the immune system against immunogenic cancer antigens such as tumor associated antigens (TAAs) and tumor specific antigens (TSAs) (Chi et al., 2020; Luo et al., 2020). Cancer vaccines have already shown promising results in clinical trials against solid cancers such as melanoma as well as breast, cervical and prostate cancer (Donninger et al., 2021).

In PC, however, only limited success has been observed so far, despite various different vaccination strategies currently in clinical trials. The major reason for vaccination failure in PC is the high variability of TAAs, which generally limits the induction of an efficient anti-tumor response, in combination with a largely immunosuppressive TME. Additionally, vaccines can often induce tumor-specific immune tolerance through Tregs (Fan et al., 2020; King et al., 2022).

In recent years, nucleic acid-based vaccination platforms have gained growing attention because they are inexpensive, carry inherent immunogenicity, and can be easily adapted (Qin et al., 2021). Nucleic acid-based vaccines, particularly mRNA, typically have to be delivered using an NP carrier such as lipid NPs. A DNA vaccine targeting the glycolytic enzyme and plasminogen receptor α-enolase (ENO1) was studied in two mouse models of PC, Kras(G12D)/Cre (KC) mice and Kras (G12D)/Trp53 (R172H)/Cre (KPC) mice. ENO1 DNA vaccination elicits humoral and cellular immune responses against tumors and delayed tumor progression, leading to significantly increased survival (Cappello et al., 2013; Cappello et al., 2018). Cancer vaccines based on mRNA have been successfully investigated in preclinical and clinical studies on various types of cancer, but only very few (pre) clinical studies focus particularly on PC (Huang et al., 2021; He et al., 2022). Nevertheless, a phase I clinical trial is currently being conducted to evaluate the safety and tolerability of mRNA5671/V941 when used as a monotherapy or in combination with pembrolizumab for patients with advanced or metastatic PC (NCT03948763) (Miao et al., 2021).

It remains to be seen whether current limitations of clinical effectiveness using vaccines for PC can be overcome in combination with ICIs. As with CAR T cell therapy, vaccination strategies are likely to be similarly impaired by the immunosuppressive and physical properties of the PC TME. Thus, targeting components within the TME is crucial in view of increasing the effectiveness of both immunotherapies as well as drug delivery. Recent advances in nanomaterials may serve as important tools to enable such targeting and are outlined in the following sections.

## Clinical application of nanoparticle-mediated targeting in pancreatic cancer immunotherapy

### Nanoparticle-based targeting strategies

**FIGURE 2:**
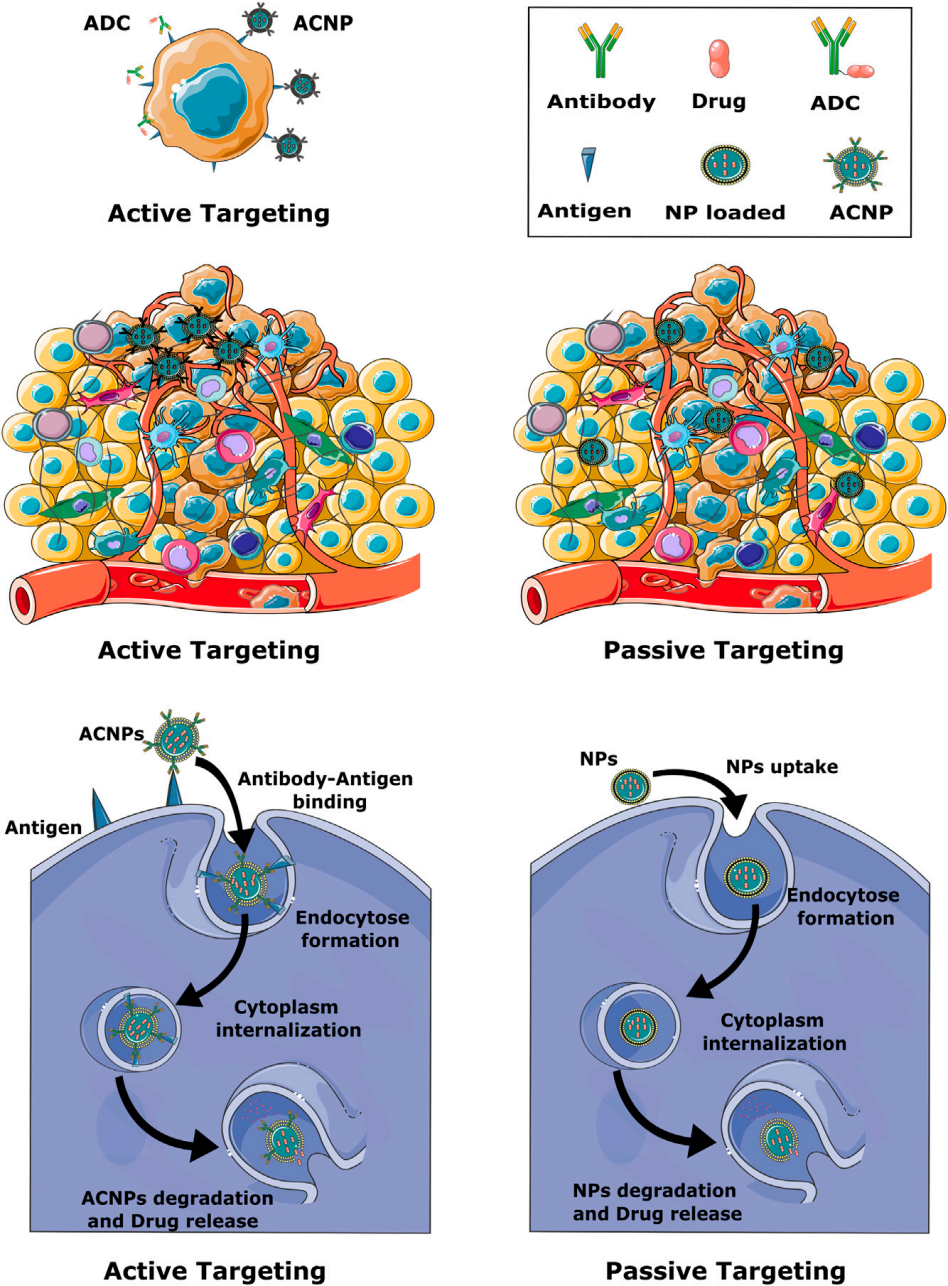
Schematic representation of active and passive targeting mechanisms. Active targeting is based on the presence of antibodies on the surface of the NP. In passive targeting, NPs mainly diffuse through leaky blood vessels and accumulate in the tumor tissue due to the EPR effect, while active targeting promotes the accumulation of NPs in the immediate vicinity of tumor cells. Formation of an antibody-antigen complex on the surface of cancer cells greatly facilitates endocytosis of the nanoparticles.

NP-dependent cancer immunotherapy is based on different targeting strategies, including NPs that can directly target cancer cells, the TME or components of the peripheral immune system. Generally, targeting can be established *via* active or passive mechanisms (Figure 2) (Navya et al., 2019; Sun et al., 2020).

Due to the unique characteristics of solid cancers, including abnormal lymphatic function and defective vasculature, NPs are able to accumulate in cancer sites by an enhanced permeability and retention effect (EPR). This passive targeting mechanism allows the effective delivery of NPs to the tumor site but does not facilitate penetration of the cancer cells. Active targeting is based on the specific interaction. Thus, in active targeting, the surface of the NPs is either coated with a ligand or an antibody that recognizes cellular receptors or components of the TME (Figure 2). Active targeting can be used in combination with passive targeting, reinforcing the activity of the accumulated NPs. As a result, drugs carried in NPs have a prolonged duration at the cancer site and side effects are reduced (Alavi and Hamidi, 2019; Attia et al., 2019; Patel and Patel, 2019).

Most chemotherapy-carrying NPs currently on the market do not specifically target tumor cells. They reach the cancer site merely on the basis of leaky vasculature and accumulate on the basis of the EPR effect. Therefore, antibody-conjugated nanoparticles (ACNPs), as a method of targeted drug delivery in cancer treatment, has been discussed to be a more effective approach to eradicate cancer cells (Johnston and Scott, 2018; Attia et al., 2019). The concept of ACNPs is closely related to antibody-drug conjugates (ADCs). ADCs are mainly mAbs with a drug or toxin payload bound to the Fc region with the help of a linker, which enables targeted drug delivery (Figure 2) (Khongorzul et al., 2020; Etrych et al., 2022). To effectively deliver the drug to the tumor, three conditions must be met: firstly, the tumor cells must overexpress the target antigen in order to engulf the ADC; secondly, the side effects must be low, so the linker between the payload and the antibody must be insensitive; and thirdly, the ratio between drug and antibody must be optimally adjusted (Etrych et al., 2022). An analogous strategy is utilized by ACNPs, with the obvious difference that the transported drug is encapsulated in or bound to NPs. Compared to ADCs, ACNPs display higher drug-to-antibody ratios and can therefore deliver higher concentrations of therapeutic agents to cancer cells (Johnston and Scott, 2018; Juan et al., 2020a). Moreover, drug delivery can be better controlled with ACNPs, and toxicity of the drug is effectively reduced while preserving its chemical properties, since the environment is shielded from the drug transported within the NP. Similar to ADC, ACNPs bind to the target cells *via* antibody-antigen interactions. The ACNPs is then absorbed by an endosomal transport mechanism and the drug can be released into the cells (Figure 2) (Juan et al., 2020b). The above principles of nanoconjugate design could substantially increase the effectiveness of anti-cancer therapies for PC, be it in either targeting TME components and tumor-infiltrating cells or increasing the effectiveness of ICIs. These aspects are highlighted in the following sections.

#### Targeting of growth factors and growth factor receptors

A variety of growth factors are secreted into the TME of PC in large amounts and promote the accumulation of the ECM. NPs targeting growth factor receptors such as vascular endothelial growth factor (VEGF) have therefore shown increased efficacy in cancer therapy (Muntimadugu et al., 2017; Yao et al., 2020). The VEGF family consists of four members: VEGF-A, VEGF-B, VEGF-C, and VEGF-D. VEGF-A is involved in most physiological and pathological processes, including the regulation of angiogenesis, vascular permeability and cell migration (Garcia-Sampedro et al., 2021). The receptors for VEGF-A are VEGFR1 and VEGFR2, where VEGFR2 has a low affinity for VEGF-A but, after binding to VEGF-A, the intracellular tyrosine kinase activity is ten times higher than for VEGFR1. VEGF-A, and VEGFR-2 are involved in mediating angiogenesis in tumor growth (Garcia-Sampedro et al., 2021). The anti-VEGF-A mAb bevacizumab, has already been approved for the treatment of colorectal and renal carcinoma (Hurwitz et al., 2004; Summers et al., 2010; Rosen et al., 2017). It is being combined with other therapies such as gemcitabine and atezolizumab, in phase Ib/II clinical trials for the treatment of PC (NCT03193190). Additionally, studies are being conducted to investigate the use of NPs targeting the VEGF signaling pathway in cancer therapy, including PC (Ambasta et al., 2011; Roacho-Perez et al., 2021).

TGF-β regulates various cellular, physiological and developmental processes as well as tissue homeostasis and repair. Impairment of TGF-β leads to many pathologies, including cancer, fibrosis and immune diseases (Shen et al., 2017; Liu et al., 2021). TGF-β is often overexpressed in PC and correlates with an advanced cancer state, rapid cancer growth, metastasis and, most importantly, poor prognosis (Shen et al., 2017). Clinical trials investigated the effect of the TGF-β inhibitor galunisertib, either alone or in combination with other therapeutic strategies, in the treatment of advanced or metastatic PC (Melisi et al., 2018; Melisi et al., 2021). Galunisertib as a monotherapy was found to display an increased probability of survival for PC patients (Gueorguieva et al., 2019). In combination with gemcitabine or durvalumab, overall survival was improved compared to gemcitabine or durvalumab alone (Melisi et al., 2021). Recently, a bifunctional TGF-β antagonist/IL-15 protein was enrolled in a phase Ib/II clinical trial and will soon start recruitment (NCT05304936). In addition, LY364947, a TGF-β inhibitor, was used in a mesoporous silica-based NP to reduce pericyte coverage of cancer vessels and thus enhance the delivery of gemcitabine, contained in a liposome-based NP, in a mouse model of PC which resulted in decreased tumor growth (Meng et al., 2013).

The epidermal growth factor receptor (EGFR), which is involved in cell differentiation, migration as well as cell growth, plays an important role in cancer growth and therapy resistance. Highly expressed in various solid cancers, including breast, colon, lung and PC, the EGFR promotes rapid mitosis and stamina in cancer cells (Shetty et al., 2021). Erlotinib, which was the first targeted chemotherapy drug to be approved for the treatment of metastatic PC, is an EGFR tyrosine kinase inhibitor (Grapa et al., 2019; Ayati et al., 2020). Recently, amivantamab, an anti-EGFR mAb, was approved by the FDA for the treatment of non-small cell lung cancer (NSCLC). In addition, a phase II clinical trial is ongoing to evaluate the toxicity and efficacy of a bispecific antibody targeting EGFR for the treatment of PC (NCT03269526) (Syed, 2021). An ADC containing an anti-EGFR antibody conjugated to a toxic agent, monomethyl auristatin E, has been developed and already demonstrated to be effective in killing PC cells (Li et al., 2019). Furthermore, an ACNPs therapy based on cetuximab, an anti-EGFR conjugated with an NP encapsulating the topoisomerase inhibitor camptothecin, showed a reduction in cancer growth in PC cell lines (McDaid et al., 2019).

#### Targeting of tumor-infiltrating cells

In addition to the above, the TME can be modulated by direct targeting of different immune cells, thereby manipulating the release of cytokines and chemokines involved in immunosuppression. NPs directly targeting T cells, dendritic cells (DCs) and TAMs have been considered as a potential strategy for cancer immunotherapy (Qian et al., 2018). Additionally, anti-cancer responses could be enhanced by ICI of NCRs either expressed on T cells, professional antigen presenting cells or cancer cells (Qian et al., 2018; Guevara et al., 2020; Yang et al., 2021). Drug delivery by NPs can also be used to modulate or reactivate dendritic cells and TAMs. *In vitro*, monocytes can either be differentiated into either M1, also called classical macrophages, with pro-inflammatory and anti-cancer activities, or M2, also called alternative macrophages, with anti-inflammatory and pro-cancer activities. TAMs often resemble an M2 phenotype and release anti-inflammatory cytokines and growth factors which promote cancer development. NPs directed at TAMs have the potential to reprogram the polarization of M2 macrophages in order to recruit M1 macrophages to cancer sites and thus enhance the pro-inflammatory and anti-cancer responses in the TME (Yang et al., 2021). In an orthotopic allograft model of PC, an interfering double-stranded RNA encapsulated in lipid calcium phosphate NPs induced the overexpression of pro-inflammatory cytokines and thus promoted polarization into M1 macrophages in the TME (Das et al., 2019). In addition, blocking the recruitment of monocytes to the tumor by targeting the CC-chemokine ligand 2 (CCL2)–CC-chemokine receptor, showed a reduction in tumor growth and less metastasis in a mouse model of PC (Sanford et al., 2013; Hu et al., 2019). In addition to macrophages, dendritic cells can be targeted by ACNPs *via* specific markers such as CD40 or CD11c triggering their activation and maturation. The activated and matured DCs stimulate T cells and thus facilitate anti-cancer immunity (Dominguez and Lustgarten, 2010; Cruz et al., 2014). In a mouse model of B16 melanoma, gold NPs with a modified form of cytosine-phosphorothioate-guanine oligodeoxynucleotides that elicit IgG responses were used to trigger DC activation (Lin et al., 2013). Moreover, an NP-based polyethylenimine mixed with the MDA5 ligand poly (I:C) promoted the recruitment of DCs to the PC microenvironment in a mouse model (Duewell et al., 2015).

The ability of NP-based strategies to modulate cells within the TME to promote their anti-cancer functions rather than immunosuppression is of particular importance to PC therapy in view of overcoming the current limitations of CAR T cell therapy and vaccination approaches mentioned earlier in this review.

#### Targeting of immune checkpoints

As mentioned earlier, ICs play an important role in healthy tissue, such as preventing autoimmune diseases and maintaining immune homeostasis. However, cancer cells exploit this mechanism to evade immune responses. ICI therapy alone has shown limited success but is at the same time associated with significant side effects in cancer, as their immunomodulatory effect is not limited to the tumor site. Thus, a combination with the targeting capabilities of NPs has attracted considerable attention (Tang et al., 2022).

Synergistic administration of an antibody targeting PD-1 and a CpG oligodeoxynucleotides loaded in mesoporous silica NPs successfully prevented cancer recurrence (Gupta et al., 2021). Similarly, a nanoparticle-based synergistic strategy was suggested that combines *in situ* vaccination using CpG and gene-mediated anti-PD therapy. In a tumor-bearing mouse model an amplified T cell response, together with enhanced NK cell infiltration were observed after this combined treatment (Hu et al., 2021b). Furthermore, local and targeted cancer immunotherapies using microneedles to deliver NPs were successfully tested in a melanoma mouse model. Here, microneedle patches containing dextran NPs loaded with anti-PD-1 antibodies and glucose oxidase were used to deliver checkpoint inhibitors to melanoma in a pH-dependent manner (Wang et al., 2016; Han et al., 2020). Moreover, a mouse breast cancer model using BMS-202 small molecule inhibitor of the PD-1/PD-L1 pathway loaded to NPs showed excellent results, as did anti PD-L1 drugs alone, including suppression of primary and distant tumor growth and attack as well as destruction of metastasized tumor cells (Zhang et al., 2019). In another study, near-infrared radiation combined with doxorubicin- and anti-PD-L1-conjugated gold NPs were shown to be effective against colorectal tumor cell lines by inhibiting cell proliferation and thus promoting apoptosis (Emami et al., 2019). Hyaluronic acid composite NPs, modified with 1-methyl-DL-tryptophan, a molecule that inhibits indoleamine-2, 3-dioxygenase, and loaded with an anti-PD-1 antagonist, were effective in a melanoma mouse model (Ye et al., 2016). In a different approach, NP encapsulating CTLA-4 siRNA showed a decrease in CTLA-4 expression and a suppression of tumor growth after systemic administration in a B16 melanoma mouse tumor model (Li et al., 2016). A phase I clinical trial to confirm the appropriate dosage of an mRNA encoding human OX40L, IL-23, and IL-36γ loaded LNP is currently underway for the treatment of solid tumor recurrence (NCT03739931). Additionally, as reviewed by Deal and colleagues, mRNA-encoded antibodies have also been developed for LNPs (Deal et al., 2021). This strategy allow LNP formulations to be used as a platform for the delivery of mRNA encoding -bi-specific antibodies or cytokines, called RiboMabs or Ribocytokines. Bispecific RiboMabs can target CD3 and TAAs and therefore bind T cells to cancer cells, leading to improved T cell-mediated antitumor activity (NCT04710043) (Guevara et al., 2020). Clinicals trials of LNPs encapsulating antibody-coding mRNA are currently being conducted in phase 1 and 2 to assess the preliminary efficacy and the appropriate dose of the drug (NCT05262530 and NCT04455620).

An siRNA targeting PD-L1 (siPD-L1) encapsulated in poly(lactic-co-glycolic acid) (PLGA) NP was recently used in a humanized mouse model of PC, effectively suppressing PD-L1 expression and resulting in a significant reduction in tumor progression (Jung et al., 2021). Similarly, in a syngeneic murine PC model, magnetic NPs loaded with siPD-L1 in combination with gemcitabine showed a decrease in tumor volume of more than 85% after 2 weeks of treatment, while all control groups treated with gemcitabine alone were moribund after 6 weeks (Yoo et al., 2019). In addition, an enhancement of activated CD8 TILs and low PD-L1 expression was observed. Magnetic resonance imaging (MRI) was used to observe the response to treatment (Yoo et al., 2019). Recently, in a mouse model of PC, assembled NPs composed of cyclodextrin-grafted hyaluronic acid and adamantine-conjugated heterodimers of pyropheophorbide a and JQ1, a PD-L1 inhibitor, showed promising results in overcoming resistance to PC treatment (Sun et al., 2021). Furthermore, the TGF-β receptor inhibitor LY2157299 encapsulated in NPs and siPD-L1 accumulated on the NP surface, enhanced anti-tumor activity in the subcutaneous Panc02 xenograft mice model by silencing PD-L1 gene expression in tumor cells and promoting CD8^+^ T cell proliferation, resulting in suppression of tumor growth (Wang et al., 2020b). In a 3D cell culture model of metastatic PC, the PD-/PD-L1 inhibitor BMS-202 was loaded into albumin NPs and encapsulated in liposome NPs sensitive to the fibrotic matrix. These NPs showed an enhanced anti-tumor response triggered by reactivated T cells and complemented by a significant release of inflammatory cytokines such as TNF-α and IFN-γ (Yu et al., 2021). In this regard, using NPs as drug carriers could be an effective approach to increase the efficacy of the ICI therapy for PC and overcome the resistance to conventional cancer therapy (Liu et al., 2022).

Despite the optimistic outcome observed by blocking ICs in immunotherapies of metastatic cancer, the effect on metastatic PC has been relatively moderate. This is largely due to its unique TME, rich in matrix remodeling fibroblasts and immunosuppressive cells (Gupta and Kim, 2021; Yu et al., 2021). In addition, various adverse effects such as skin rashes and arthritis have been observed with the use of these drugs. Targeted delivery with NPs has shown potential to reduce toxicities and to prolongs retention time in the tumor tissue. Thus, the TME and tumor cells can be directly and extensively targeted (Yang et al., 2021). Moreover, since the advent of the first chemotherapies, immunotherapies are currently considered as the most important new development in cancer treatment strategies (Yoo et al., 2019). For this reason, NPs, as potent carriers for ICIs in PC immunotherapies, have attracted increasing interest.

## Conclusion and outlook

**FIGURE 3:**
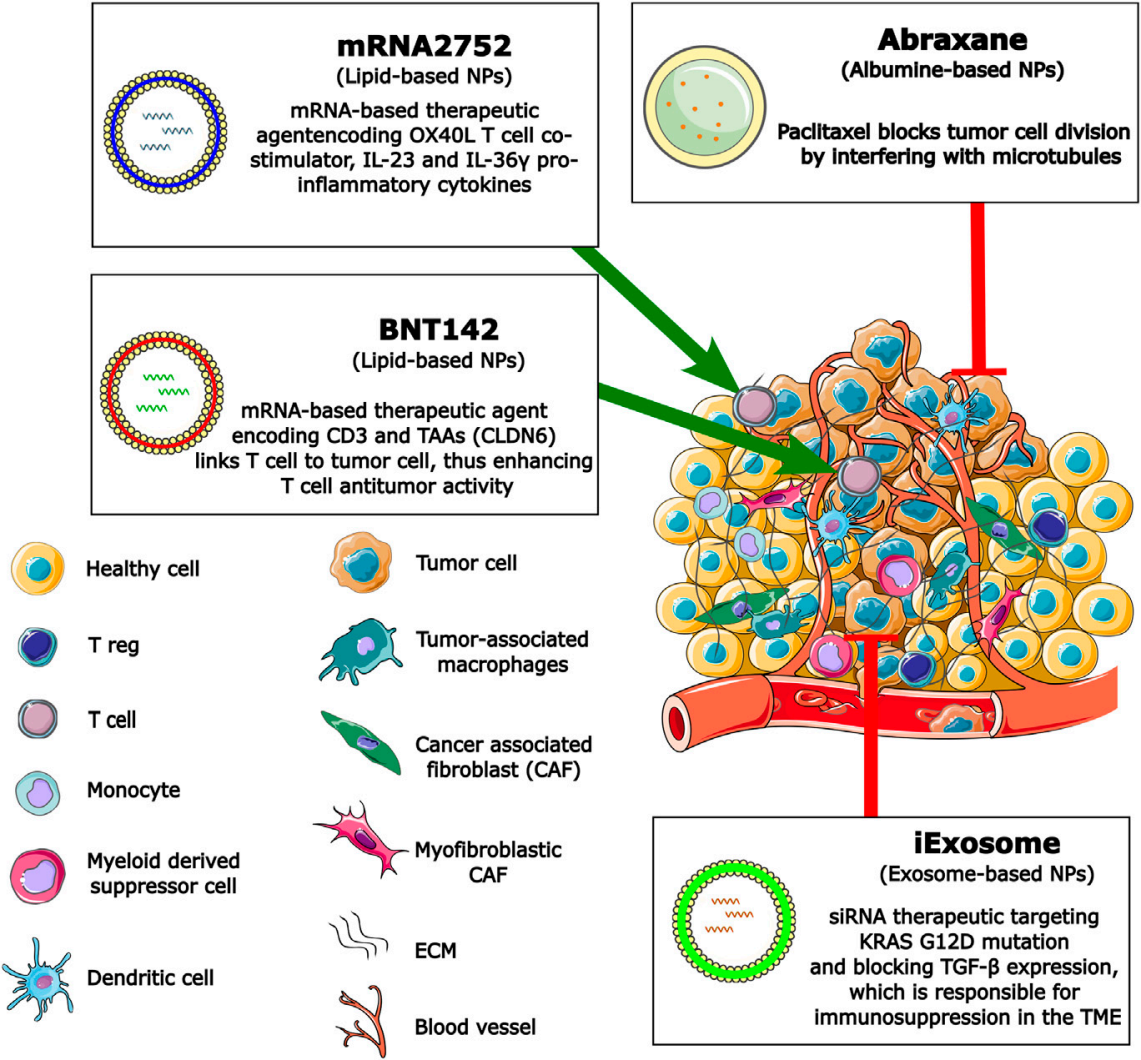
Schematic representation of NP strategies used to overcome and remodulate the TME or reactivate antitumor T cell activities in PC.

PC remains one of the deadliest cancers with limited treatment options for the vast majority of patients. It is expected to become a major cause of cancer deaths over the next 20 years, especially as the number of new cases of PC increases year on year (Hu et al., 2021a). Treatment and survival have increased significantly for most cancers in recent years, especially due to developments in immunotherapy. Recent advances suggest that NP-based therapies may further increase the efficacy of PC therapy. Effectiveness of other main cancer therapies, including chemotherapy and radiotherapy, is limited mainly due to the unique TME in PC. Recently, therapy with ICIs has shown promising results in several solid cancers such as lung cancer and melanoma. The results were less promising in PC, which highlights the urgent and unmet need for new therapeutic strategies for patients with unresectable PC. Targeting chemotherapy with NPs has shown optimistic, albeit moderate, results in PC, but NP therapy targeting the TME components or infiltrating cells in PC is under development and showing promising results in preclinical studies (summarized in Figure 3).

NP drug delivery strategies are widely used in tumor therapy. It should be noted that most trials using NP strategies in PC are based on chemotherapy (Table 1), despite the fact that chemotherapy has largely been ineffective and has significant adverse events. The development of novel tumor therapies using NP-based immunotherapy is therefore urgently required for the treatment of PC, as well as for other cancers. Generally, chemotherapy has also shown excellent results when used in combination with ICI therapy in research trials. However, in the case of PC, there are almost no studies concerning ICI therapy delivered by NPs alone (Table 2), although some studies have examined the NP-based ICI delivery in combination with chemotherapy in animal models or PC cell lines with substantial anti-tumor effects. The combination of immunotherapy and other therapeutic strategies in the administration of NPs indicates more promising results (Table 3). Therefore, it would be useful to design strategies for the delivery of NPs which overcome the TME of PC by directly targeting its components and infiltrating cells to reactivate anti-tumor activity, as well as using toxins carried by NPs which directly target tumor growth. Recent developments in NP design have hopefully now put the elusive, but crucial, goal of more effective therapies for PC within reach.

**TABLE 1:** Clinical trials of NP-based chemo- or radiotherapy for pancreatic cancer.

Chemotherapeutic or radiotherapeutic							
Name	Type of NP	Drug formulation	Type of therapy	Phase	Results	Clinical trials	Completion date
Abraxane	Albumin	Paclitaxel Albumin-Stabilized NP	Chemotherapy	2	6-months OS of 58%; Median PFS: 1.7 months	NCT00691054	Dec. 2012
EndoTAG-1	Liposome	Paclitaxel + Gemcitabine	Chemotherapy	3	not available	NCT03126435	Oct. 2021
Abraxane	Albumin	Paclitaxel Albumin-Stabilized NP + Gemcitabine	Chemotherapy	2	not available	NCT04115163	Dec. 2021
SNB-101	Core-Shell biopolymer	SN-38 active metabolite of irinotecan	Chemotherapy	1	ongoing	NCT04640480	March 2022
Abraxane	Albumin	Paclitaxel Albumin-NP + Gemcitabine Hydrochloride + Irinotecan Hydrochloride	Chemotherapy	2	2 years OS of 47%–48%; Median PFS 10.9–14.2 months; Study is ongoing	NCT02562716	Oct. 2022
ThermoDox^®^	Heat-sensitive Liposome	doxorubicin	Chemotherapy	1	ongoing	NCT04852367	Dec. 2022
Abraxane	Albumin	Paclitaxel Albumin-NP + Gemcitabine Hydrochloride + Metformin Hydrochloride	Chemotherapy	1	ongoing	NCT02336087	Dec. 2022
Imx-110	PEG-PE	Stat3/NF-kB/poly-tyrosine kinase inhibitor and low-dose doxorubicin	Chemotherapy	1 and 2	ongoing	NCT03382340	July 2023
Abraxane	Albumin	Paclitaxel Albumin-NP + Hydroxychloroquine + Paricalcitol + Gemcitabine	Chemotherapy	2	ongoing	NCT04524702	Aug. 2023
Abraxane	Albumin	Paclitaxel Albumin-NP + Gemcitabine + Telotristat Ethyl	Chemotherapy	2	ongoing	NCT03910387	Oct. 2023
Abraxane	Albumin	Paclitaxel Albumin-NP + Bosentan + Gemcitabine	Chemotherapy	1	ongoing	NCT04158635	Dec. 2023
AGuIX	Gadolinium	Magnetic Resonance Radiation	Radiotherapy	1 and 2	ongoing	NCT04789486	Sept. 2024
Onivyde	Liposome	Irinotecan Sucrosofate + Fluorouracil + Leucovorin Calcium + Rucaparib	Chemotherapy	1 and 2	ongoing	NCT03337087	Aug. 2025

**TABLE 2:** | Clinical trials of NP-based immunotherapy.

Immunotherapeutic							
Name	Types of NPs	Drug formulation	Type of therapy	Phase	Results	Clinical trials	Completion date
V941	Lipid	mRNA-5671/V941 alone/plus Pembrolizumab (PD-L1-Inhibitor)	Cancer Vaccine	1	ongoing	NCT03948763	Aug. 2022
mRNA-2752	Lipid	mRNA-2752 alone/plus Durvalumab (PD-L1-Inhibitor)	Immuno-modulation	1	ongoing	NCT03739931	Jan. 2023
BNT152/153	Lipid	mRNA-encoded IL-7, IL-2	Immuno-modulation	1	ongoing	NCT05262530	Oct. 2023
BNT142	Lipid	mRNA-encoded CD3 + CLDN6	Immuno-modulation	1 and 2	ongoing	NCT04710043	April 2026
BNT151	Lipid	mRNA-encoded Optimized IL-2	Immuno-modulation	1 and 2	ongoing	NCT04455620	June 2026

**TABLE 3:** Clinical trials of different NP-based targeted or combined therapies.

Targeted or combined therapies							
Name	Types of NPs	Drug formulation	Type of therapy	Phase	Results	Clinical trials	Completion date
TKM 080301	Lipid	siRNA Against the PLK1 Gene Product	PLK1 inhibitors	1	not available	NCT01437007	June 2012
NBF-006	Lipid	siRNA against Glutathione S-Transferase P	Glutathione S-Transferase P inhibitors	1	ongoing	NCT03819387	March 2023
iExosome	Exosome	Mesenchymal Stromal Cells-derived Exosomes with KRAS G12D siRNA	KRAS inhibitors	1	ongoing	NCT03608631	March 2023
RO7198457	Lipid	RO7198457 + Atezolizumab + mFOLFIRINOX	Immuno/Chemotherapy	1	ongoing	NCT04161755	Nov. 2023
Abraxane	Albumin	Paclitaxel Albumin-Stabilized NP + Durvalumab + Oleclumab + Gemcitabine	Immuno/Chemotherapy	2	ongoing	NCT04940286	Oct. 2024
BNT141	Lipid	mRNA-encoded antibodies + Nab-paclitaxel + Gemcitabine	Immuno/Chemotherapy	1 and 2	ongoing	NCT04683939	Sept 2024
NBTXR3	Hafnium Oxide	NBTXR3 + Radiation	Chemo/Radiotherapy	1	ongoing	NCT04484909	Dec. 2026
